# Supplementary material for: Content-rich biological network constructed by mining PubMed abstracts
Source: BMC Bioinformatics. 2004 Oct 8;5:147. doi: 10.1186/1471-2105-5-147 (PMC528731; doi:10.1186/1471-2105-5-147)
Supplement: Additional File 2 — The original results of the above study (non-essential files are deleted to keep the file size under the limit set by BMC bioinformatics). [file 1471-2105-5-147-S2.bz2 › chilibotAdditionalFile2/dip10/290ID1833185E1120/html/CDC2_CCNB1.html]

 


 **CDC2** and **CCNB1** 
  
Found 825 abstracts in PubMed, retrieved 10.  
 

 What does Google say? 
 PDF only 
| .edu only 

---

**Interactive relationship** (e.g. stimulation, inhibition, etc)

**Inhibitory relationship**- Thus, the impairment in cell cycle progression could not be explained by a lack of cyclin B1  [ **CCNB1** ] , or the ability of  **Cdc2**  and cyclin B1  [ **CCNB1** ]  to associate, but instead resulted, at least in part, from impaired  **Cdc2**  activity.  Ref: 12883482 Hepatology, 2003
**Neutral relationship**- Immunoblot analysis revealed that ethanol metabolism had little affect on total  **Cdc2**  content in these cells, but resulted in the accumulation of up to 20 times the amount of cyclin B1  [ **CCNB1** ] , indicating that cyclin B1  [ **CCNB1** ]  was available for formation of  **Cdc2**  cyclin B1  [ **CCNB1** ]  complexes.  Ref: 12883482 Hepatology, 2003
- **Cdc2**  is positively regulated by association with cyclin B1  [ **CCNB1** ] , and negatively regulated by phosphorylation of amino acids Thr14 and Tyr15.  Ref: 12883482 Hepatology, 2003

**Non-interactive relationship** (e.g. studied together, co-existance, homology, etc.)

- Genetic analysis of fission yeast and physiological studies of vertebrate and invertebrate oocytes showed that activation of cyclin B  [ **CCNB1** ]   **cdc2**  kinase triggers mitosis.  Ref: 12836412 Med Sci Paris , 2003
- Our results show that the mRNA and protein levels of the G 2 M regulators  **cdc2** , cyclin B1  [ **CCNB1** ]  and cdc25C are down regulated in MCF7TDX cells, while unaffected in MCF7 cells.  Ref: 12853888 Anticancer Drugs, 2003
- Finally, forced expression of the NF YA protein in cells committed to differentiate leads to an impairment in the down regulation of cyclin A, cyclin B1  [ **CCNB1** ] , and cdk1  [ **CDC2** ]  expression and is accompanied by a delay in myogenin expression.  Ref: 12857858 Mol Biol Cell, 2003
- Co immunoprecipitation revealed that 6 times more  **Cdc2**  cyclin B1  [ **CCNB1** ]  complexes were present in the ethanol treated cells compared with the controls.  Ref: 12883482 Hepatology, 2003
- This was associated with earlier expression of the cell cycle promoting cyclin A2, cyclin E, cyclin B1  [ **CCNB1** ] , cyclin F and cyclin dependent kinase 1  [ **CDC2** ]  Cdk 1  [ **CDC2** ]  genes and diminished protein levels of Cdk  [ **CDC2** ]  inhibitor p21Cip1.  Ref: 12867420 J Biol ChemJ Biol Chem, 2003
- The gene expression of cyclin B  [ **CCNB1** ]  and p34cdc2  [ **CDC2** ]  was increased after the 1 day treatment, reverted to the control level at 2 and 12 weeks of treatment, and was remarkably increased after the 52 week treatment.  Ref: 12841865 Cancer Sci, 2003
- p21 Waf1 Cip1 exerts a key role in driving this G 2 exit both by inhibiting cyclin B1  [ **CCNB1** ]  Cdk1  [ **CDC2** ]  and cyclin A Cdk1  [ **CDC2** ]  2 complexes, which control G 2 M progression, and by blocking the phosphorylation of pRb family proteins.  Ref: 12881433 EMBO JEMBO J, 2003
- cyclin D1, cyclin B  [ **CCNB1** ] , and p34cdc2  [ **CDC2** ]  were examined by RT PCR.  Ref: 12841865 Cancer Sci, 2003
- We have previously found that the overexpression of p53 causes G 2 arrest and represses the synthesis of cyclin dependent kinase 1  [ **CDC2** ]  and cyclin B1  [ **CCNB1** ] , two proteins required for cells to traverse from G 2 into M.  Ref: 12874009 Cancer Res, 2003
- A stronger G2 M arrest by these combinations was accompanied by a substantial decrease in the levels of  **cdc2** , cyclin B1  [ **CCNB1** ]  and cdc25C.  Ref: 12866029 Int J CancerInt J Cancer, 2003
